# The Dietary Antioxidant Piceatannol Inhibits Adipogenesis of Human Adipose Mesenchymal Stem Cells and Limits Glucose Transport and Lipogenic Activities in Adipocytes

**DOI:** 10.3390/ijms19072081

**Published:** 2018-07-17

**Authors:** Christian Carpéné, Héctor Pejenaute, Raquel del Moral, Nathalie Boulet, Elizabeth Hijona, Fernando Andrade, Maria Jesùs Villanueva-Millán, Leixuri Aguirre, José Miguel Arbones-Mainar

**Affiliations:** 1 INSERM U1048, Institute of Metabolic and Cardiovascular Diseases (I2MC), Paul Sabatier University, 31059 Toulouse, France; nathalie.boulet@inserm.fr; 2 Adipocyte and Fat Biology Laboratory (AdipoFat), Unidad de Investigación Traslacional, Instituto Aragonés de Ciencias de la Salud (IACS), Instituto de Investigación Sanitaria (IIS) Aragon, 50009 Zaragoza, Spain; fles@usj.es (H.P.); email@adipofat.com (R.d.M.); 3 Department of Gastroenterology, University of Basque Country (UPV/EHU), Biodonostia Research Institute, 20014 San Sebastián, Spain; ELIZABETH.HIJONA@biodonostia.org; 4 Division of Metabolism, Cruces University Hospital and BioCruces Health Research Institute, Plaza de Cruces s/n, 48903 Barakaldo, Spain; FERNANDO.ANDRADELODEIRO@osakidetza.eus; 5 HIV and Associated Metabolic Alterations Unit, Infectious Diseases Department, Center for Biomedical Research of La Rioja (CIBIR), 26006 Logroño, Spain; chusmillan85@gmail.com; 6 Nutrition and Obesity Group, Department of Nutrition and Food Science, Faculty of Pharmacy and Lucio Lascaray Research Center, University of the Basque Country (UPV/EHU), 01006 Vitoria, Spain; leixuri.aguirre@ehu.eus; 7 CIBEROBN Physiopathology of Obesity and Nutrition, Institute of Health Carlos III (ISCIII), 28029 Madrid, Spain

**Keywords:** stilbenoids, fat cells, adipogenesis, resveratrol, hydrogen peroxide

## Abstract

Phenolic compounds are among the most investigated herbal remedies, as is especially the case for resveratrol. Many reports have shown its anti-aging properties and the ability to reduce obesity and diabetes induced by high-fat diet in mice. However, such beneficial effects hardly translate from animal models to humans. The scientific community has therefore tested whether other plant phenolic compounds may surpass the effects of resveratrol. In this regard, it has been reported that piceatannol reproduces in rodents the anti-obesity actions of its parent polyphenol. However, the capacity of piceatannol to inhibit adipocyte differentiation in humans has not been characterized so far. Here, we investigated whether piceatannol was antiadipogenic and antilipogenic in human preadipocytes. Human mesenchymal stem cells (hMSC), isolated from adipose tissues of lean and obese individuals, were differentiated into mature adipocytes with or without piceatannol, and their functions were explored. Fifty µM of piceatannol deeply limited synthesis/accumulation of lipids in both murine and hMSC-derived adipocytes. Interestingly, this phenomenon occurred irrespective of being added at the earlier or later stages of adipocyte differentiation. Moreover, piceatannol lowered glucose transport into adipocytes and decreased the expression of key elements of the lipogenic pathway (PPARγ, FAS, and GLUT4). Thus, the confirmation of the antiadipogenic properties of piceatanol in vitro warrants the realization of clinical studies for the application of this compound in the treatment of the metabolic complications associated with obesity.

## 1. Introduction

Obesity and its complications have reached epidemic proportions, being considered as serious public health threats worldwide. Considering the limited results of classical pharmacological approaches for the treatment of obesity, phytochemicals have emerged as a novel alternative to mitigate its morbidity. Natural phenolic compounds, such as resveratrol, are among the most studied anti-obesity phytochemicals since 2006, when it was reported that resveratrol had anti-aging properties and was able to reduce obesity and diabetes induced by high-fat diet in mice [1]. Those beneficial effects were partially confirmed in other animal studies as well as in humans (reviewed in [2]). Nevertheless, recent meta-analyses converged to indicate that the effects were limited and that there is still insufficient evidence to support the use of resveratrol supplements for the management of obesity in humans [3,4]. Even the extreme term “resveratrol fiasco” has been coined to reflect this scientific deception [5]. As a consequence, the research for resveratrol-like molecules with higher bioavailability and equivalent, or even greater, anti-obesity properties has been the object of intense efforts recently [6].

Piceatannol (trans-3,4,3′,5′-tetrahydroxystilbene) is a natural phenolic stilbenoid chemically similar to resveratrol but having an extra hydroxyl group at the 3′ position. It is present in various kinds of food such as berries, passion fruit seeds, peanuts, grapes, wine, and white tea [7]. Although piceatannol has been the subject of far less numerous studies than resveratrol [8], its effects on limiting hypercholesterolemia, atherosclerosis, arrhythmia, and angiogenesis, which are common complications of obesity, have already been described [9]. Regarding adipose tissue physiopathology, it has been repeatedly shown that piceatannol inhibits adipogenesis in 3T3-L1 cells, a murine model of preadipocytes [10,11,12]. Similar antiadipogenic effect has been observed on a stable cell line derived from human liposarcoma [13]. Although all those cell lines are useful models for the study of differentiation into adipocytes, one should keep in mind that their metabolism (and mainly their lipid handling) might deviate from the physiological metabolism of mature adipocytes found in the adipose tissue [14]. This prompted us to verify whether such potential antiadipogenic action was effective in cells isolated from human non-pathological fat depots.

In this study, we used human adipocytes derived from adipose mesenchymal stem cells (hMSC) to investigate the effect of piceatannol on the adipogenic process and the metabolic status of the adipocytes. Our results confirm that previous observations of the antiadipogenic properties of piceatannol in preadipocyte lineages can be extrapolated to human adipocytes.

## 2. Results

### 2.1. Piceatannol Impairs Lipid Accumulation and Inhibits Lipogenesis in Mouse Adipocytes

**Figure 1:**
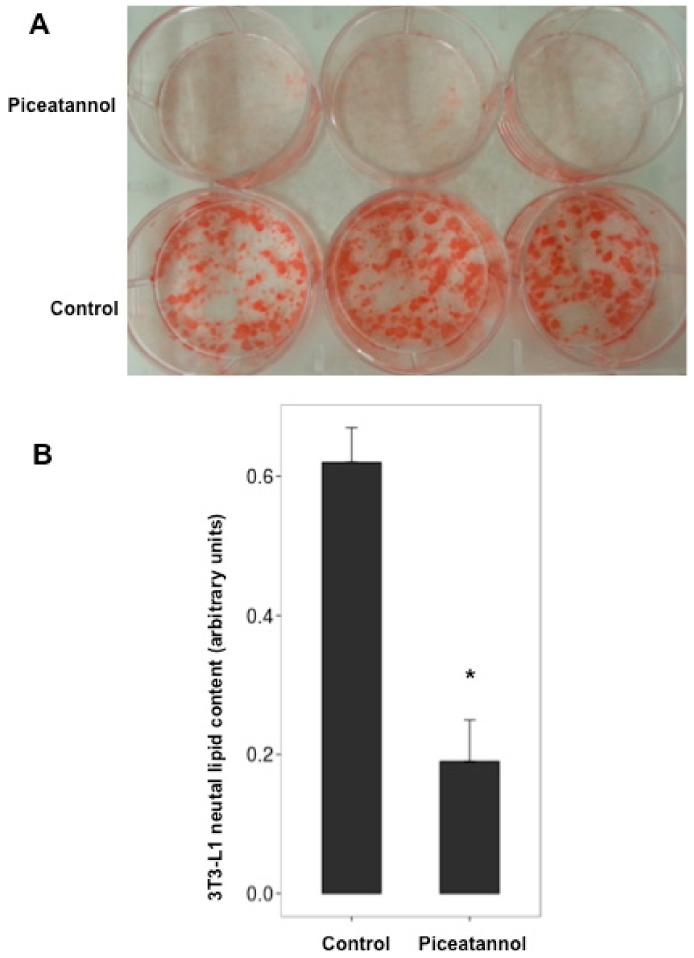
Influence of piceatannol on lipid accumulation in 3T3-L1 derived adipocytes. (**A**) Oil Red O staining of the mature adipocytes cultured in multi-well plates after differentiation without (control) or with 50 µM piceatannol. (**B**) Quantitative analysis of neutral lipid content. Mean ± SEM of six determinations. Different from control at * *p* < 0.05 by Student’s *t*-test.

**Figure 2:**
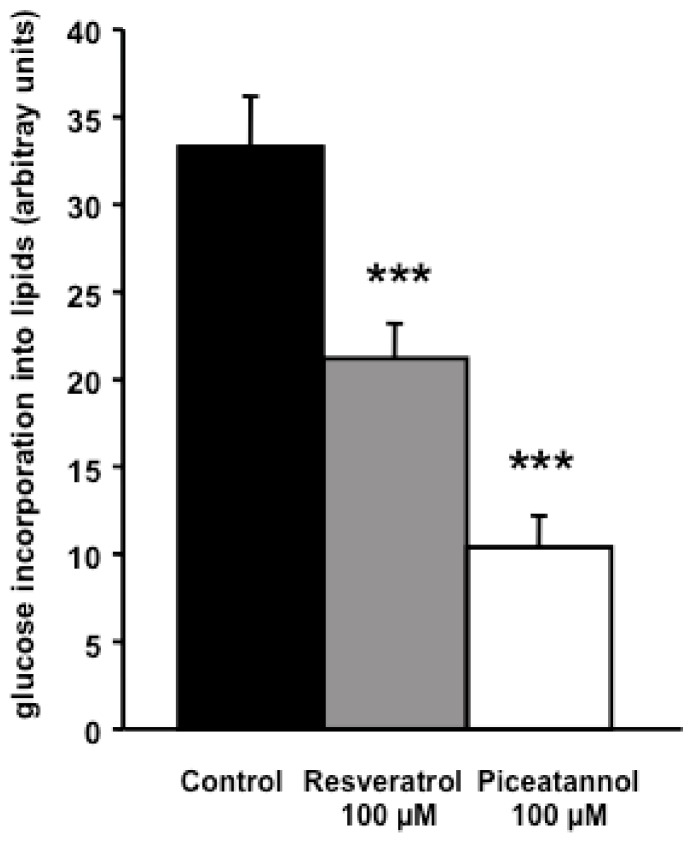
Short-term inhibition by piceatannol and resveratrol of the incorporation of glucose into intracellular lipids in mouse adipocytes. Radiolabelled glucose was incubated for 2 h at 37 °C with freshly prepared mouse adipocytes in the absence (control, black column) or the presence of 100 µM polyphenol, then radiolabelled lipids were extracted and counted. Glucose incorporation into lipids was expressed in units relative to the maximal lipogenic effect of insulin. Mean ± SEM of 14 determinations. *** *p* < 0.001 for the differences between the control, resveratrol, and piceatannol treated groups by one-way ANOVA and Dunnett’s post-hoc test.

Post-confluent 3T3-L1 preadipocytes were treated for one week with piceatannol during the differentiation process. 50 µM piceatannol produced a 65% reduction of the neutral lipid content in mature adipocytes (Figure 1A,B), which account for more than 90% of the lipids in this in vitro model [15]. This preliminary experiment confirmed previous observations indicating that piceatannol is as antiadipogenic as other phenolic compounds when tested in mouse preadipocytes [10,11,12]. To further extend our verification of the inhibitory effect of this natural molecule on lipid accumulation, we tested whether piceatannol and its parent polyphenol, resveratrol, were able to limit the incorporation of radiolabelled glucose into lipids in mature mouse adipocytes. For this, mouse adipocytes were incubated with 0.1 mM [^3^H]-glucose and a 100 µM dose of each polyphenol for 120 min. The incorporation of radiolabelled glucose into newly synthesized lipids was more reduced by piceatannol than by resveratrol (Figure 2) in a relatively short-term manner. Thus, we obtained a clear confirmation of the capacity of piceatannol to rapidly inhibit de novo lipogenesis in mouse adipocytes.

### 2.2. Piceatannol Impairs Adipogenesis in Human Preadipocytes

**Figure 3:**
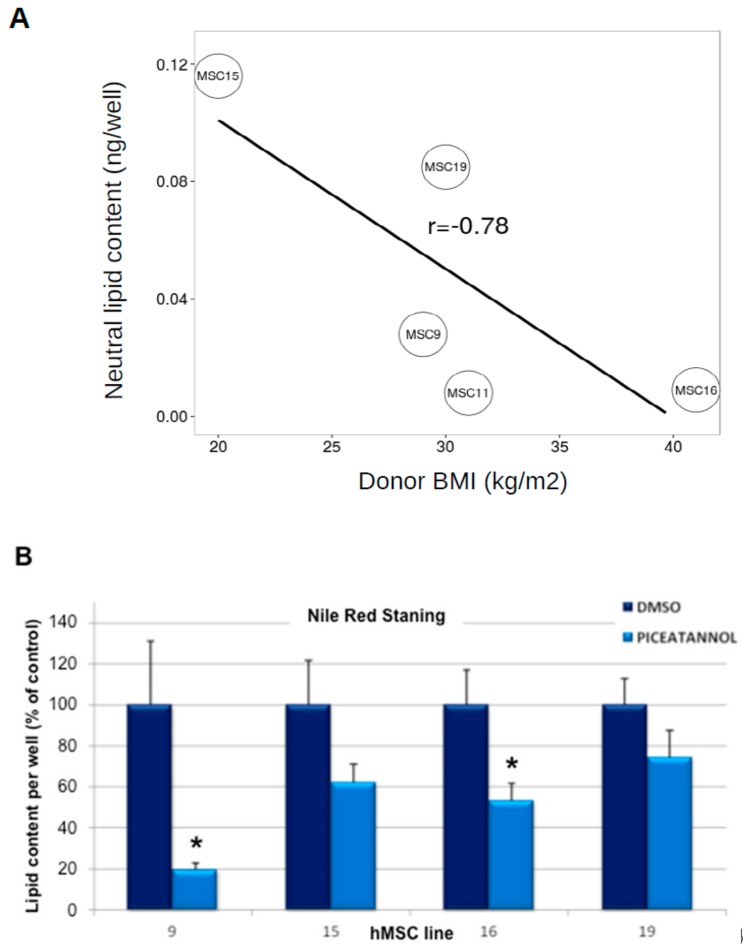
Influence of piceatannol on lipid accumulation in hMSC-derived adipocytes. hMSC obtained from five individuals anonymously renamed MSC 9, 11, 15, 16, and 19 were differentiated into adipocytes. (**A**) Relationship between body mass index of donor and lipid accumulation per well. Each point on the Y-axis is the mean of quadruplicates. Significant coefficient of linear regression is indicated. (**B**) Effect of 50 µM piceatannol on lipid accumulation in four hMSC lines expressed as percentage of optimal adipogenic condition in the presence of vehicle (DMSO, dark columns). Each percentage is the mean ± SEM of eight replicates. Different from vehicle at * *p* < 0.05 by Student’s *t*-test.

First, we sought to rule out a cytotoxic effect of piceatannol and treated hMSC lines once differentiated into adipocytes with piceatannol-supplemented medium for 48 h. No noticeable effect on cell viability was observed in the assayed range (0–50 μM) of piceatannol (not shown). Thereafter, we verified whether the inter-individual variability observed in the human donors, at least regarding their body mass index (BMI), was recapitulated in hMSC-derived adipocytes. To this end, different hMSC lines were established from five different donors, and the lipid accumulation obtained in hMSC-derived adipocytes was measured with the lipophilic stain Nile Red. This easy-to-use marker of the ability of hMSC to differentiate into adipocytes indicated that such in vitro capability was negatively associated with the BMI of the donor. A strongly impaired differentiation of hMSCs into adipocytes was observed as donor’s BMI increased, probably reflecting an exhausted pool of hMSC in the adipose tissue of the obese donors (Figure 3A). This comparative approach showed that both MSC11 and MSC16 cell lines had similar very low levels of lipid accumulation and prompted us to exclude MSC11 from further studies.

We then investigated the effect of piceatannol on the process that drives hMSC to accumulate lipids, hereafter called adipocyte differentiation or adipogenesis. During this experiment, four lines of hMSC were treated with the standard differentiation cocktail supplemented with either 0 or 50 μM piceatannol for seven days. Subsequent lipophilic staining with Nile Red showed that piceatannol produced a reduction of lipid-containing cells in all the hMSC lines differentiating into adipocytes, when compared to their respective untreated control (Figure 3B). The inhibitions of 80%, 38%, 47% and 26% of lipid storage in each cell line indicated that the anti-adipogenic properties of the resveratrol derivative were effective in human cells.

**Figure 4:**
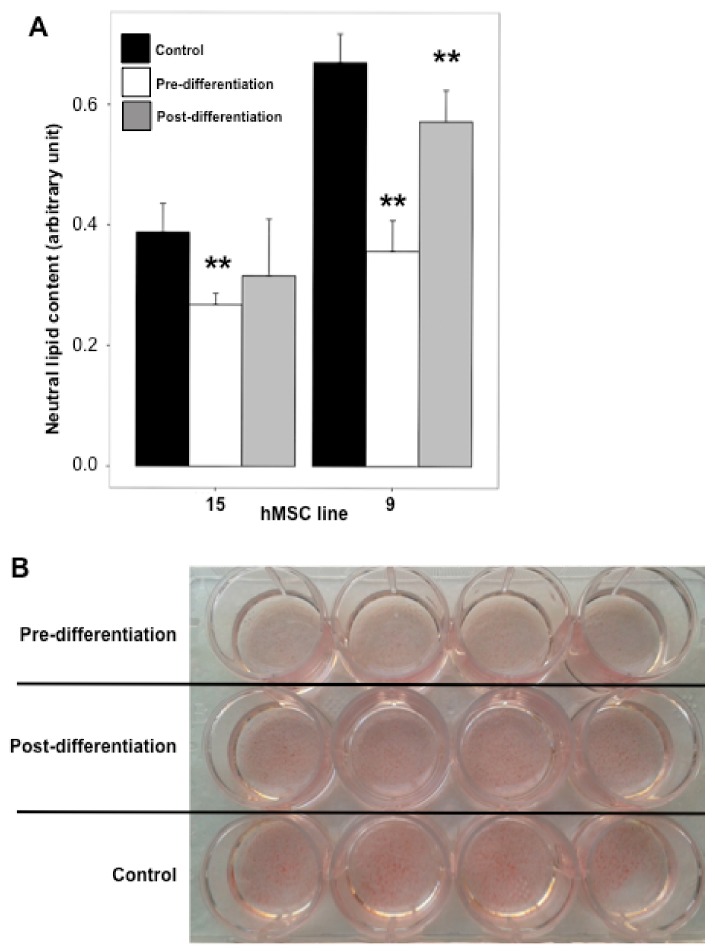
Influence of piceatannol on lipid accumulation during the early and late phase of in vitro adipogenesis. (**A**) Neutral lipid content in control (black column) or hMSC treated by medium supplemented with 50 µM piceatannol before (pre-differentiation, white column) of after induction of adipogenesis (post-differentiation, shaded column). Each column is the mean ± SEM of quadruplicates for the indicated hMSC lines. ** *p* < 0.01 for the differences between the control and the pre- and post-differentiation treatments by one-way ANOVA and Dunnett’s post-hoc test. (**B**) Picture of a multi-well culture plate with lipid staining of the line hMSC 9 after control and pre- and post-differentiation treatment with 50 µM piceatannol.

In another set of experiments, mature (eight days post-differentiation) hMSC-adipocytes were treated with either 0 or 50 μM piceatannol for seven additional days, and intracellular lipids were determined in two lines. Again, a reduction was observed in piceatannol-treated cells, though less pronounced than when cells of the same donors were treated during the differentiation process as above described (Figure 4A). Briefly, the reduction in lipid accumulation was approximately of 50% when treating the cells during all the adipogenic process, while it was ~15% when piceatannol was applied to already differentiated adipocytes (Figure 4B). Based on our observations, it can be deduced that piceatannol impairs adipogenesis in human adipocytes in a maturation-dependent fashion.

**Figure 5:**
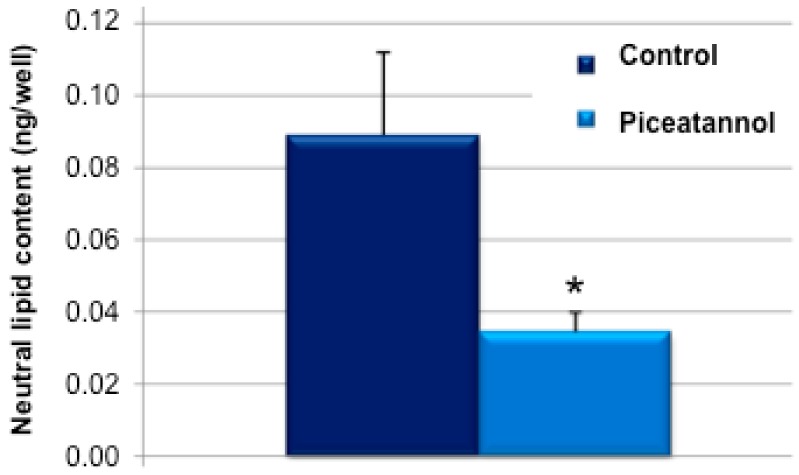
Inhibition by piceatannol of lipid accumulation in human adipocytes. Each column represents the mean ± SEM of a total of 48 determinations made on a total of four hMSC lines. Difference between 50 µM piceatannol and control condition (DMSO vehicle) at * *p* < 0.05 by Student’s *t*-test.

To illustrate the overall antiadipogenic effects of piceatannol we pooled the delipidating effects obtained in the 4 hMSC lines. Figure 5 shows an ~60% average reduction of cellular lipid content when hMSCs where differentiated into adipocytes in the presence of 50 µM piceatannol.

### 2.3. Piceatannol Induces Desensitization to Insulin Activation of Glucose Uptake and Down-Regulates Lipogenic Enzymes in Human Adipocytes

**Figure 6:**
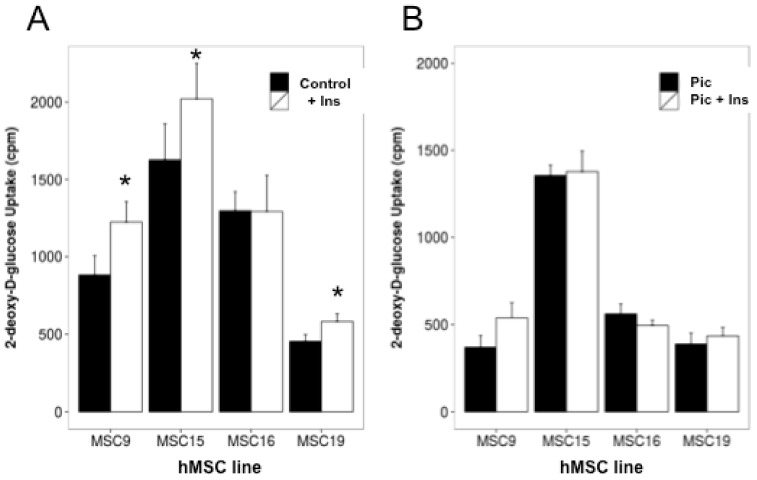
Effect of piceatannol on hexose uptake by differentiated hMSC-adipocytes. (**A**) Stimulation of [^3^H]-deoxyglucose uptake by 500 nM insulin in four hMSC lines. The basal hexose uptake determined under baseline conditions (control, black columns) was increased by insulin (Ins, open columns) in three of the four lines. Different from respective control at * *p* < 0.05 by Student’s *t*-test. (**B**) Impairment by piceatannol treatment (Pic) during adipocyte differentiation of the hexose uptake stimulation by insulin. There was no difference between Pic and Pic + Ins conditions. Each column is the mean ± SEM of six replicates.

Glucose uptake is the first step of glucose utilization and its inhibition may lead to impaired de novo lipogenesis (DNL). We therefore, examined the effect of piceatannol on basal and insulin-stimulated glucose transport activity in mature hMSC-adipocytes. Insulin increased [^3^H]-deoxyglucose uptake compared to baseline. Such stimulation was significant in at least three of the four lines tested (Figure 6A). This insulin action was hampered when adipocytes were treated with 50 µM piceatannol during their differentiation process (Figure 6B). In addition, the piceatannol also reduced basal glucose uptake in two of the four lines studied: MSC9 and MSC16. Although the latter was apparently insensitive to insulin (not so unexpectedly since obtained from a donor with BMI > 40), it can be assessed that piceatannol was altering either basal or stimulated glucose transport in all the lines studied.

**Figure 7:**
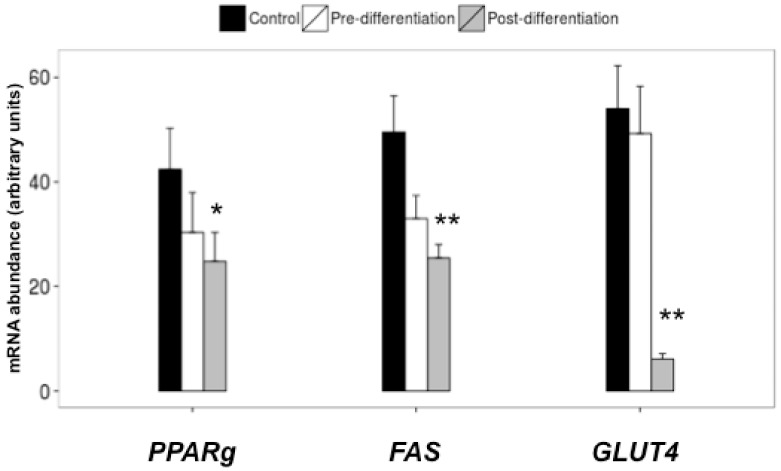
Effect of piceatannol on gene expression of major markers of adipocyte differentiation. The mRNA abundance is expressed for control, pre- and post-differentiation treatment with 50 µM piceatannol, as relative units. Each column corresponding to the indicated gene is the mean ± SEM of four hMSC lines. * *p* < 0.05 and ** *p* < 0.01 for the differences between the control the pre- and post-differentiation treatments by one-way ANOVA and Dunnett’s post-hoc test.

Thus, supplementation with piceatannol blunted any increment of glucose transport into the cells under adipogenic culture conditions, ultimately depriving the adipogenic enzymes of the building blocks necessary to produce fatty acids and accumulate triglycerides. Consistent with these results, piceatannol-treated hMSC-adipocytes showed lower mRNA levels of the adipogenic regulators PPARγ and FAS (Figure 7). Moreover, the expression of glucose transporter GLUT4, which emerges during adipogenesis, was down-regulated by the polyphenol when compared to non-treated counterparts (Figure 7).

Such impairment by piceatannol of various steps of the lipogenic pathway, observed in adipocytes derived from human adipose, totally fits with previous observations made on mouse adipocytes. Moreover, we also observed that the expression of adiponectin, an adipokine specifically produced by insulin-sensitive adipocytes, was dramatically reduced by piceatannol treatment (not shown), indicating that, alongside the lipogenic pathway, many other aspects of the adipogenic differentiation process were altered.

### 2.4. Piceatannol Dose-Dependently Limits Hydrogen Peroxide Detection in Human Adipose Tissue Preparations

**Figure 8:**
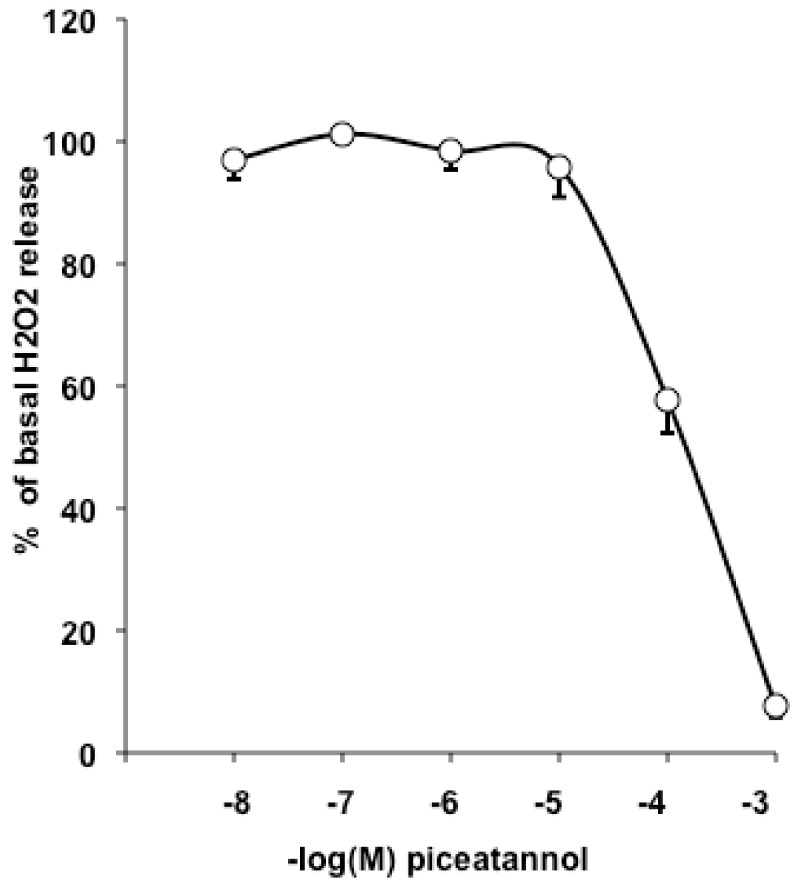
Inhibition of hydrogen peroxide detection by piceatannol in human adipose tissue (AT) preparations. Mean ± SEM of five individuals.

Insulin stimulation increases hydrogen peroxide production by NADPH oxidase in adipocytes [16]. In turn, hydrogen peroxide may mimic insulin activation of the glucose transport and lipogenic activities [17]. Consequently, we next tested whether the polyphenol could modify the fate of hydrogen peroxide release in preparations of human adipose tissue. Piceatannol reproduced in human subcutaneous adipose tissue (AT) the known antioxidant properties of stilbenoids, since it inhibited almost totally the spontaneous release of hydrogen peroxide (Figure 8). Although piceatannol was ineffective between 10^−8^ and 10^−5^ M, it clearly lowered the detected levels of hydrogen peroxide release when present at 10^−4^ or 10^−3^ M in the AT homogenates for 30 min.

## 3. Discussion

Our in vitro study accumulated evidences of diverse antiadipogenic effects of piceatannol. First, as an antioxidant, it rapidly altered in AT the fate of hydrogen peroxide, an agent known to activate glucose transport and lipogenesis in adipocytes [17,18]. Second, piceatannol rapidly abolished the glucose incorporation into lipids in mouse adipocytes, as already reported for two related stilbenoids—resveratrol and pterostilbene [19]. Third, when present during hMSC differentiation, it clearly prevented lipid accumulation, impaired insulin stimulation of glucose uptake and limited the expression of important genes in the adipogenic programme.

The dose of 50 µM piceatannol used for chronic treatments in this study was indicated as optimal in the pioneering work of Kwon and coworkers [10], showing that at this dose, piceatannol clearly inhibited adipogenesis in murine preadipocytes. However, this dose might not be the lower limit of piceatannol effectiveness regarding lipid deposition. We have observed in a recent study that the inhibition of triglyceride content in 3T3-L1 differentiating preadipocytes was detectable with 25 µM of the molecule, while 10 µM was clearly insufficient to alter adipogenesis [11]. A major limitation of our study is that it cannot be assessed whether 50 µM piceatannol is a concentration reachable in the adipose depots after nutritional intervention. The plasma values reported during pharmacokinetic experiments in animal models led to consider that the upper limit for circulating piceatannol could be set at approximately 10 µM [20]. Hence, the clinical relevance of piceatannol supplementation in mitigating obesity must be evaluated with caution, although piceatannol did appear to be more active than resveratrol at micromolar doses in reducing lipid deposition in mouse adipocytes.

Several clinical trials with this natural polyphenol have been already performed while other are under completion. Among these recent controlled supplementations, the work reported by Kitada and colleagues [21] indicated that oral piceatannol administration (purified from passion fruit seed extract) does not promote weight loss, but improves several complications of obesity, such as elevated insulin levels, high blood pressure, and elevated heart rate in men with BMI ≥ 25. A protective effect of piceatannol on 3T3-L1 adipocytes exposed to the proinflammatory molecule TNFα has also been described [22]. In contrast, our in vitro data indicate that piceatannol affects negatively both basal and insulin-stimulated glucose transport in hMSC differentiated into adipocytes. Even when there was practically an absolute resistance to insulin action in cells derived from one of the donors, piceatannol treatment further hampered glucose uptake. Such inhibition of insulin responsiveness induced by previous exposure of human adipose cells to piceatannol appears however in agreement with its inhibitory role for the tyrosine kinase activity of the insulin receptor in 3T3-L1 models [10], similarly to resveratrol [23]. Since during this study we could not obtain freshly prepared adipocytes from subcutaneous AT of donors, it was not possible to directly evidence whether piceatannol can rapidly inhibit lipogenic activity of human adipocytes ex vivo. Nevertheless, as we already reported that hydrogen peroxide mimics several insulin actions in human adipocytes [18], we posit that the inhibitory influence of piceatannol on the fate of hydrogen peroxide in the AT could be mirrored by an inhibition of adipocyte lipogenic activity.

Piceatannol was recently proposed to inhibit glyceraldehyde 3-phosphate dehydrogenase (GAPDH), the enzyme catalyzing the 6th step of glycolysis, and known to exert many other functions. It is the piceatannol capacity to interact with the highly reactive cysteine residue in the enzyme’s active site that prompted the authors to propose a redox-sensitive inhibition [24]. However, this GAPDH enzyme is not the same as the glycerol-3-phosphate dehydrogenase (GPDH), which catalyzes the reversible conversion between dihydroxyacetone phosphate and glycerol 3-phosphate. This GPDH is an important step in the lipogenic pathway located at the crossroad between carbohydrate and lipid metabolism. As is the case with GAPDH, one isoform of GPDH is located in the cytoplasm coupled to a NADH/NAD+ exchange, we hypothesize that GAPDH might be potentially affected by piceatannol, too. Such putative mechanism may reconcile the rapid and long-lasting inhibitory effects of piceatannol on lipid storage in fat cells, since the observed down-regulation of GLUT4, which fits with long-lasting anti-adipogenic action, cannot on its own explain the rapid impairment of glucose incorporation into lipids observed within two hours in mouse adipocytes.

Regarding piceatannol’s capacity to lower the detection of hydrogen peroxide in AT preparations, it should be noted that the antioxidant effects of piceatannol occurred at concentrations similar than that required for the antilipogenic effect but higher than that limiting adipogenesis. This does not indicate whether the antioxidant capacity of piceatannol is necessary or sufficient for its antilipogenic/antiadipogenic properties. Regarding antioxidant capacity on its own, it has been recently reported that piceatannol is ~10 times more effective than resveratrol in scavenging reactive oxygen species [25]. Those impressive antioxidant properties of piceatannol might be the consequence of a more “equilibrated” molecule [26,27] since it possesses two hydroxyl groups on each of its two phenolic cycles, while resveratrol contains only a total of three OH. We had previously reported that piceatannol inhibits MAO activity [27], a mitochondrial enzyme that generates hydrogen peroxide and which is highly expressed in human adipocytes [28], suggesting that piceatannol cannot be considered as a mere antioxidant and reinforcing the idea that adipocytes might belong to the list of its targets.

Piceatannol’s delipidating properties have been gaining traction since it was reported to limit fat accumulation in murine preadipocyte cell lines by two independent studies in 2012 [10,13]. Its anti-adipogenic action was later confirmed in the same model [11,12], and extended to the LiSa-2 cells [13] derived from a human liposarcoma [29]. More recently, it has been reported that piceatannol can also limit fat deposition in *Caenorhabditis elegans* [30] and in mice fed a high-fat diet [31]. Additional reports have also been published on piceatannol’s ability to lower circulating lipids in the spontaneously obese Zucker rats [11] and in high-fat diet fed Fisher rats [32], although without reducing their body weight gain. Thus, piceatannol, structurally similar to resveratrol but less studied for its anti-obesity actions, exhibits potential interest to limit the development of fat stores in vivo. We conclude that piceatannol can act, among other targets, upon the adipocytes and their precursors within fat depots. This in vitro study paves the way for further research to support whether piceatannol supplementation in humans may function as a novel anti-obesity approach in the context of an integrated weight loss or preventive strategy.

## 4. Materials and Methods

### 4.1. Patients and Human Adipose Tissue Samples

hMSC were obtained after collagenase digestion of subcutaneous abdominal fat from patients undergoing bariatric surgery at the Hospital Universitario Miguel Servet (Zaragoza, Spain) following a previously described protocol [33], which was approved by the Clinical Investigation Ethics Committee of Aragon (ref #11/2013, approval date: 6 December 2013). A total of five donors were selected from a cohort for their different body mass index (BMI), varying from 20 to >40. One was excluded after the first preliminary tests for not fulfilling the criterion of apparent lack of metabolic dysfunctions, as the excluded individual was inadvertently on antidiabetic medication.

Samples of human subcutaneous adipose tissue were obtained from five overweight women undergoing reconstructive surgery at Rangueil Hospital (Toulouse, France) who gave their informed consent for inclusion before they participated in the study. Their mean age was 43 years, and mean body mass index was 25.6 ± 0.8 kg/m^2^. Biopsies were kept at −80 °C without any medium. The study was in compliance with the INSERM guidelines and approved by the local ethic committee (reference DC-2008-452)

### 4.2. Cell Cultures and Treatments

Cells were maintained at 37 °C in a humidified 5% CO_2_ atmosphere, and cultured until confluence in DMEM (Lonza, Verviers, Belgium) containing 10% fetal bovine serum (FBS), 4 mM glutamine, 1 mM sodium pyruvate, and 1% penicillin/streptomycin. The concentration of glucose for this growing phase was 1 g/L for hMSC and 4.5 g/L for 3T3-L1 cells. Post-confluent differentiation of hMSC or 3T3-L1 was induced with DMEM 4.5 g/L glucose (Lonza, Verviers, Belgium) supplemented with 10% FBS, 1 μM dexamethasone, 0.5 mM isobutylmethylxanthine, 1 μM rosiglitazone, and 1.67 μM human insulin. This medium was changed every 72 h until cells were differentiated in the case of hMSC. In the case of 3T3-L1 preadipocytes the challenge to this differentiating medium lasted 2 days, then the cells were changed to same medium with insulin only. Differentiating cells were grown in 6- or 12-well plates, and media supplemented with either vehicle (0.06% DMSO) or with piceatannol (Selleckchem, Houston, TX, USA) at 50 µM final. Two protocols of treatment were performed for the hMSC: one, named “pre-differentiation,” lasted one week with piceatannol, followed by one week with high-glucose culture medium; while the other, named “post-differentiation,” consisted in one week with differentiation medium, followed by one week in high-glucose medium supplemented with piceatannol.

At the end of treatments, several wells were used for cytotoxicity determination while in other wells, supernatant was removed, and cells were used for triglyceride determination, RNA extraction, or glucose uptake assays.

### 4.3. Assessment of Cell Viability, Lipid Accumulation, and Expression of Lipogenic Enzymes

Cell viability was assessed using the neutral red assay (TOX4 kit, Sigma-Aldrich, St. Louis, MO, USA) following manufacturer’s recommendations.

The culture wells of hMSC or 3T3-L1 were fixed with 1 mL of 10% paraformaldehyde in PBS for 15 min. Then, 400 μL of Oil Red O solution were added before microphotography with a Nikon Eclipse TS100 microscope (Nikon Corporation, Tokyo, Japan). Lastly, the dye was extracted by 100% isopropanol and optical density was quantified at 500 nm. Alternatively, Nile Red staining was performed similarly save that fluorescence readouts were detected at 485/590 nm.

RNA samples were extracted from cells treated or not with piceatannol by using Trizol (Invitrogen, Carlsbad, CA, USA), according to the manufacturer’s instructions. The integrity of the RNA extracted was verified using RNA 6000 Nano Assay (Thermo Scientific, Wilmington, DE, USA). RNA samples were then treated with DNaseI kit (Applied Biosystems Inc., Foster City, CA, USA) to remove genomic DNA. Reverse transcription was performed on 1 µg of total RNA, and relative mRNA levels of enzymes involved in adipocyte differentiation and lipogenic pathways were quantified using real-time PCR and the primer sequences as already described [34].

### 4.4. Glucose Transport and Incorporation into Lipids

When hMSC were considered as fully differentiated, two weeks after confluence, they were incubated in 600 μL/well of KRPH medium (136 mM NaCl, 5 mM NaH_2_PO_4_, 4.7 mM KCl, 1 mM MgSO_4_, 1 mM CaCl_2_ and 20 mM HEPES) during 90 min at 37 °C and 5% CO_2_, then treated without or with insulin (0.5 μM) for additional 30 min. The addition of 0.30 μCi/well of [^3^H]-Deoxy–d-Glucose (1 mCi/mL, #NET549250UC, Perkin-Elmer), for 10 min corresponded to the hexose uptake assay, which was achieved by extensive washing with KRPH and by lysing the cells in 500 μL of 0.2 N NaOH in 0.1% SDS at 4 °C. Aliquots of 300 μL were mixed with 2 mL of scintillation liquid (Ultima Gold, PERKIN-ELMER #6013321), and the incorporated mixture was radioactivity counted in a 1209 Rackbeta Liquid Scintillation Counter (LKB Wallac Co., Turku, Finland).

Incorporation of d-[3-^3^H]-glucose into lipids was assessed in mouse fat cells according to a recently described protocol [35], then validated in [19]. To this aim, 14 C57Bl6 mice of both gender were obtained from Charles River, L’arbresle, France), housed in plastic cages and sacrificed in accordance with INSERM guidelines, to remove intra-abdominal fat pads and separate adipocytes as recently reported [36]. Briefly, freshly isolated adipocytes were incubated at 37 °C in the same medium as above, except that energy was provided by d-[3-^3^H]-glucose at 0.6 mM final concentration in vials that were used for 120-min incubation and for lipid extraction by non-water miscible liquid scintillation cocktail (InstaFluorPlus, PerkinElmer, Waltham, MA, USA). Then, only the radiolabelled glucose that was incorporated in neo-synthesized radioactive lipids (organic phase) was counted in the same device as for glucose uptake assay.

### 4.5. Hydrogen Peroxide Detection in Adipose Tissue Homogenates

Homogenates were prepared just prior assays from thawed hAT samples by using homogenizer Tissue Master-125 (Omni International, Kennesaw, GA, USA) for approximately 40 sec. Using 50 µL of homogenates distributed in 96-well black microplates, the chromogenic mixture was added to reach 4 U/mL horseradish peroxidase and 40 μM 10-acetyl-3,7-dihydrophenoxazine in a final volume of 200 µL of 200 mM phosphate buffer (pH 7.4). Hydrogen peroxide was measured owing to the fluorescent probe as initially described by Zhou et al. for the continuous fluorometric determination of monoamine oxidase activity [37], with already reported minor adaptations [27]. Before the incubation that lasted 30 min at 37 °C, the preparations were preincubated for 30 min alone or with piceatannol at the indicated final concentrations. The fluorescent signal (ex/em: 540/590 nm) was collected in a Fluoroskan Ascent plate reader (ThermoLabsystems, Helsinki, Finland), and the readouts after 30-min incubation were subtracted from corresponding values at time 0. Assay of hydrogen peroxide from 0.05 to 5 µM was performed in parallel and used for standard curve as previously detailed [27].

### 4.6. Statistical Analysis

Experimental data in vitro were expressed as the means ± SEM of the indicated number of experiments or replicates. We used Student’s *t* test for comparing two groups and one-way ANOVA, followed of Dunnett’s test for multiple comparisons. All statistical analysis was performed using Graph Pad Prism and R (v.3.0.3) software (https://cran.r-project.org/). The differences were deemed to be statistically significant at *p* values < 0.05.

## 5. Conclusions

To conclude, our present work gives a clinical relevance for all these observations by reporting for the first time the antiadipogenic effect and delipidating effect of piceatannol in human adipocytes derived from stroma cells. Although limited to in vitro conditions and to piceatannol, our finding indicate that the class of stilbenoids, and more widely many other phytoalexins known to act as antimicrobial compounds in plants, can be useful to prevent or treat metabolic diseases in a manner as—or even more—efficient than that of their parent molecule, resveratrol, which currently lacks strong scientific support for being considered as a novel anti-obesity agent.
